# Is pinworm infection still a public health concern among children in resource-rich regions? Trends in pinworm infection prevalence and associated factors among children in Hualien County, Taiwan: a retrospective cross-sectional study

**DOI:** 10.1186/s12889-022-14641-4

**Published:** 2022-11-28

**Authors:** Yu-Chao Hsiao, Jen-Hung Wang, Chia-Hsiang Chu, Yu-Hsun Chang, Yung-Chieh Chang, Rong-Hwa Jan, Shao-Yin Chu, Shang-Hsien Yang, Jui-Shia Chen, Ming-Chun Chen

**Affiliations:** 1 Department of Pediatrics, Hualien Tzu Chi Hospital, Buddhist Tzu Chi Medical Foundation, Hualien, Taiwan; 2 Department of Medical Research, Hualien Tzu Chi Hospital, Buddhist Tzu Chi Medical Foundation, Hualien, Taiwan; 3 School of Medicine, Tzu Chi University, Hualien, Taiwan; 4 Department of Pediatrics, National Taiwan University Hospital, Taipei, Taiwan; 5 Department of Life Science, National Dong Hwa University, Shoufeng, Taiwan

**Keywords:** Pinworm, School-age children, Trend analysis, Taiwan, Risk factors

## Abstract

**Background:**

*Enterobius vermicularis* (pinworm) is a common intestinal parasitic infection in children. A gradual decrease in the prevalence of pinworm infection has been noted in resource-rich settings, such as Taiwan. However, the influence of sociodemographic factors on the temporal trend in pinworm infection rates in children under the current pinworm infection prevention policy in Taiwan is not well characterized. This study aimed to evaluate the trend of pinworm infection prevalence and the associated factors among children in Hualien County, Taiwan.

**Methodology:**

In this retrospective cross-sectional study, we included a total of 56,197 students (aged 6–10 years) in grades 1 and 4 in Hualien in 2009–2018. Children were screened for pinworm infection using adhesive cellophane perianal swabs in the routine student health examination. Logistic regression was conducted to evaluate the factors associated with pinworm infection. Associations between dependent and independent variables were measured by odds ratios. The Cochran–Armitage test was used to assess whether there were significant trends in different stratifications. Variables with *P*-values < 0.05 were considered statistically significant.

**Results:**

A total of 56,197 school-age children from grades 1 and 4 during 2009–2018 were included. Young age and male sex were risk factors for pinworm infection (*P* < 0.001). A negative correlation between body mass index and *enterobiasis* was observed, and decreased pinworm infection was noted during the study reference period. Children living in suburban and rural areas had higher odds of having a pinworm infection than those living in urban areas (*P* < 0.001). A significant decrease in the overall prevalence rate of pinworm infection was observed among children in 2009–2018 (*P* < 0.001). However, there was no obvious change in the pinworm infection rate in rural areas during this period (*P* = 0.953), and it was higher than that in urban and suburban areas.

**Conclusions:**

The overall prevalence of pinworm infection gradually decreased from 2009 to 2018 among school-age children in Hualien. However, there was no declining trend in pinworm infection in rural areas. Young age, male sex, and rural residence were significantly associated with pinworm infection. Pinworm infection remains a major public health concern among children in rural areas of Hualien.

## Background

*Enterobius vermicularis*, also known as pinworm, is a common intestinal parasitic infection worldwide despite advances in medicine. Pinworm infection is transmitted by the fecal–oral route. When an infected person scratches the perianal area, it leads to contamination of the hand and clothing with pinworm eggs. Contact with materials or food contaminated with pinworm eggs is one of the main modes of infection. Therefore, pinworm infection easily spreads in crowded settings such as home and school. Pinworm infection is particularly common in children, especially preschool and school-age children [1]. Previous studies have identified poor hand hygiene, lower socioeconomic status, and crowded environment as risk factors for pinworm infection [2–4]. Almost half of all individuals with pinworm infection are asymptomatic or show minimal symptoms. Perianal itching, especially at night, is a common symptom, which not only impairs the sleep quality but also increases the risk of skin infection due to frequent scratching. Patients with pinworm infection may also have gastrointestinal symptoms such as chronic abdominal pain, poor appetite, or even malnutrition [5].

Pinworm infection is usually an important public issue in tropical and subtropical areas, including Taiwan. The reported prevalence of pinworm infection across the world ranges between 0.21% and 54.86% [2, 4, 6–9]. Fortunately, a gradually decreased prevalence of pinworm infection has been noted in many countries with a rigorous public health policy. For instance, studies conducted in Thailand and Germany revealed this declining trend in pinworm infection in children [4]. The prevalence of childhood pinworm infection in Taiwan declined significantly from 19.9% in 1986 to 2.5% in 2001 under concerted preventive efforts to control pinworm infection by screening school-age children with adhesive cellophane perianal swabs and treating infected children and their family members with mebendazole [10].

However, pinworm infection is still an important public health issue in some areas of resource-rich regions, regardless of the decreasing prevalence rates. A previous study conducted in Germany revealed similar findings [5]. In Taiwan, a low prevalence of childhood pinworm infection in 2008, approximately 0.62%, was reported in Taipei, the capital of Taiwan [11]. However, simultaneously, the prevalence of pinworm infection was approximately 4.2% in Hualien, which was the second-least urbanized county with 13 districts in the east of Taiwan [10]. In line with our previous study in Hualien, pinworm infection was still an important issue among school-age children in 2010 [3]. A growing body of evidence has demonstrated a significant correlation between sociodemographic factors and *enterobiasis* [12–15]. However, the influence of sociodemographic factors on pinworm infection in children residing in the same area on long-term follow-up under the current policy of pinworm prevention in Taiwan is not well characterized. To the best of our knowledge, this is the first study based on the annual health examination database of school-age children in Hualien to evaluate the trend of pinworm infection prevalence and to identify the different sociodemographic risk factors for pinworm infection among children in Hualien, Taiwan.

## Methods

### Study design, period, and area

Data for this retrospective cross-sectional study were collected from the health examinations of school-age children from 2009 to 2018 in Hualien, Taiwan. The annual health examination was conducted for students in grades 1 (aged 6–7 years) and 4 (aged 9–10 years). The annual health examination included anthropometric measurements and physical examination findings. The residential setting was categorized into three groups (urban, suburban, and rural) according to the population density, educational level, percentage of the elderly population, percentage of the agricultural population, and medical resources [16]. Hualien, the largest county in Taiwan by area, includes 13 districts with considerable differences in urbanization levels. The urban areas included Hualien City, Xincheng, and Jian, and the suburban areas included Shoufeng, Fenglin, Yuli, Guangfu, Fengbin, Ruisui, and Fuli. Rural areas included Xiulin, Wanrong, and Zhuoxi.

### Sample size and sampling technique

The study included students in grades 1 and 4 in Hualien from 2009 to 2018 who had records of their annual health examinations. Based on the parasitic infection prevention policy in Taiwan, the pinworm survey was conducted on children in grades 1 and 4. Students with missing data, such as age, sex, residential setting, body mass index (BMI), and pinworm survey, were excluded. A total of 56,197 school-age children were enrolled in this study. Males accounted for 52.3% of all subjects, and the mean ages of males and females were 8.23 ± 1.53 years and 8.27 ± 1.53 years, respectively. Most of the enrolled subjects resided in urban areas (males: 68.2%, females: 68.1%).

### Data collection and processing

In Taiwan, adhesive cellophane perianal swabs have been used to detect pinworm eggs on the anal folds [2, 10]. Pinworm eggs are easier to be detected in the affected patients than pinworms. The perianal swabs were used on two consecutive mornings by the students or their family members, and the students were asked to bring the perianal swabs immediately after completing the collection of samples. Microscopic examination was performed by experienced medical technologists, and pinworm infection was defined as the detection of pinworm eggs on microscopy [3].

Anthropometric measurements, including weight and height, were measured by school nurses prior to the dates of the physical examination. BMI was calculated, and students were classified into underweight, normal weight, overweight, and obese groups according to the age-sex-specific BMI cutoff levels in the new growth charts for Taiwanese children and adolescents, based on the standards of the World Health Organization, and health-related physical fitness related to health, developed by the Department of Health of Taiwan in 2010 [3, 17]. Demographic data in the health examination records included age, sex, and residential setting.

### Data quality control

To ensure that all the data were complete for statistical analysis, we performed data screening for all covariates to detect conditions such as missing data, data out of valid ranges, and duplicates. In the beginning, there were 57,620 records, of which 1,423 were invalid. After excluding the invalid ones, only 56,197 records were included in the data analysis.

### Data analysis

The data were coded, entered, cleaned, and analyzed using Social Science Statistical Package version 17.0 (SPSS Inc., Chicago, IL, USA), and figures showing the prevalence rates of pinworm infection in different districts of Hualien were generated using R (version 3.6.3). Descriptive statistics for sex, different BMI groups, urbanization, time periods, and pinworm infection rate were presented as frequencies or proportions. The age of students included in this study is presented as mean ± standard deviation. The Chi-squared test was used to evaluate the association of categorical variables with sex. Logistic regression was conducted to evaluate the factors associated with pinworm infection. Associations between dependent and independent variables were measured by odds ratios (OR) at 95% CI. Variables with *P*-values < 0.05 were considered statistically significant. The Cochran–Armitage test was used to assess whether there were significant trends in different stratifications.

## Results

This study was based on the annual health examination records of school-age children between 2009 and 2018. We analyzed the prevalence, trend, and associated risk factors of *E. vermicularis* infection.

Demographic information pertaining to the 56,197 subjects enrolled in this study is presented in Table 1. The overall prevalence of pinworm infection was 4.4% (2,497/56,197), and male subjects had a higher pinworm infection rate than female subjects (4.9% vs. 3.9%). Overweight and obese subjects consisted of 22.4% males and 19.2% females.

**Table 1:** Sociodemographic characteristics of study participants (*n* = 56,197)

Characteristics	Male (*n* = 29,388)	Female (*n* = 26,809)
Age (years)	8.23 ± 1.53	8.27 ± 1.53
Grade, n (%)		
1	13,919 (47.4%)	12,405 (46.3%)
4	15,469 (52.6%)	14,404 (53.7%)
BMI group, n (%)		
Underweight	6,191 (21.1%)	6,210 (23.2%)
Normal	16,617 (56.5%)	15,474 (57.7%)
Overweight	3,342 (11.4%)	2,748 (10.3%)
Obese	3,238 (11.0%)	2,377 (8.9%)
Urbanization, n (%)		
Urban	20,035 (68.2%)	18,261 (68.1%)
Suburban	6,706 (22.8%)	6,209 (23.2%)
Rural	2,647 (9.0%)	2,339 (8.7%)
Period, n (%)		
2009	3,673 (12.5%)	3,373 (12.6%)
2010	3,535 (12.0%)	3,206 (12.0%)
2011	3,077 (10.5%)	2,955 (11.0%)
2012	3,133 (10.7%)	2,708 (10.1%)
2013	2,827 (9.6%)	2,592 (9.7%)
2014	2,793 (9.5%)	2,592 (9.7%)
2015	2,487 (8.5%)	2,250 (8.4%)
2016	2,580 (8.8%)	2,339 (8.7%)
2017	2,562 (8.7%)	2,334 (8.7%)
2018	2,721 (9.3%)	2,460 (9.2%)
Pinworm, n (%)	1,450/29,388 (4.9%)	1,047/26,809 (3.9%)

Data are presented as n or mean ± standard deviation

The prevalence of pinworm infection among school-age children in Hualien from 2009 to 2018 is presented in Table 2. The overall prevalence of pinworm infection decreased from 5.2% in 2009 to 4.2% in 2018, a decrease that was statistically significant (*P* < 0.001 for the trend). To further clarify the influence of sociodemographic characteristics on pinworm infection, trend analysis was stratified by sex and urbanization. After adjusting for sex, both male and female students showed a decreased trend of pinworm rate (*P* < 0.05 for both). Urban and suburban areas reported a significant decrease from 2009 to 2018 (*P* < 0.05 for both). However, there was no obvious change in the pinworm infection rate in rural areas over 10 years (*P* = 0.953), and the rate was higher than that in urban and suburban areas.

**Table 2:** Trend analysis of the prevalence of pinworm infection stratified by gender and urbanization from 2009 to 2018

Group	Total	Pinworm rate (%)										
		2009	2010	2011	2012	2013	2014	2015	2016	2017	2018	*P*-value for trend
Overall	56,197	5.2	5.0	4.8	4.7	4.5	3.2	3.6	4.1	4.4	4.2	< 0.001*
Gender												
Male	29,388	5.7	5.7	4.9	5.2	5.2	3.6	3.9	4.8	4.8	4.9	0.004*
Female	26,809	4.7	4.4	4.7	4.2	3.6	2.8	3.2	3.2	4.0	3.5	< 0.001*
Urbanization												
Urban	38,296	4.2	3.4	3.6	3.6	2.7	2.3	2.1	2.8	3.4	3.4	0.001*
Suburban	12,915	5.5	6.3	4.6	4.0	5.2	3.2	3.8	3.5	3.7	3.2	< 0.001*
Rural	4,986	12.7	14.6	15.2	14.3	15.8	10.7	14.6	15.5	14.7	12.8	0.953

The results of multiple logistic regression analysis to identify the sociodemographic factors associated with pinworm infection in school-age children are presented in Table 3. Students of grade 1 (aged 6–7 years) and male sex were associated with significantly higher pinworm infection rates (*P* < 0.001 for both). Interestingly, a negative correlation between BMI and *enterobiasis* was observed in the study. Students in the obese (OR: 0.36) and overweight (OR: 0.52) groups had a significantly lower OR for *enterobiasis* than subjects with normal BMI (*P* < 0.001 for both). Students in the underweight group showed a trend of higher OR (1.07) for pinworm infection than children in the normal BMI group; however, the between-group difference was not statistically significant (*P* = 0.179). During the study reference period (2009–2018), decreased pinworm infection rate was noted, especially after 2013 (*P* < 0.05 from 2012 to 2018). According to the residential setting, suburban and rural areas had higher OR for pinworm infection than urban areas (suburban: 1.45, rural: 5.27;* P* < 0.001 for both), and rural areas had a higher pinworm infection rate by a factor of approximately six than urban areas.

**Table 3:** Factors associated with pinworm infection in school-age children

	Crude		Adjusted	
	Odds Ratio (95% CI)	*P*-value	Odds Ratio (95% CI)	*P*-value
Age	0.85 (0.83, 0.88)	< 0.001*	0.86 (0.84, 0.88)	< 0.001*
Gender	-	-	-	-
Male	1.28 (1.18, 1.39)	< 0.001*	1.29 (1.19, 1.40)	< 0.001*
Female	References	NA	References	NA
BMI group	-	-	-	-
Normal	References	NA	References	NA
Underweight	0.91 (0.82, 0.99)	0.043*	1.07 (0.97, 1.18)	0.179
Overweight	0.50 (0.43, 0.59)	< 0.001*	0.52 (0.44, 0.61)	< 0.001*
Obese	0.35 (0.28, 0.43)	< 0.001*	0.36 (0.29, 0.44)	< 0.001*
Year	-	-	-	-
2009	References	NA	References	NA
2010	0.96 (0.83, 1.12)	0.634	0.96 (0.82, 1.12)	0.608
2011	0.92 (0.79, 1.08)	0.320	0.91 (0.78, 1.07)	0.258
2012	0.90 (0.77, 1.06)	0.197	0.87 (0.74, 1.02)	0.091
2013	0.85 (0.72, 1.00)	0.052	0.82 (0.70, 0.98)	0.024*
2014	0.60 (0.50, 0.72)	< 0.001*	0.59 (0.49, 0.71)	< 0.001*
2015	0.68 (0.56, 0.82)	< 0.001*	0.66 (0.55, 0.80)	< 0.001*
2016	0.77 (0.64, 0.92)	0.003*	0.76 (0.63, 0.90)	0.002*
2017	0.84 (0.71, 0.99)	0.049*	0.83 (0.70, 0.99)	0.036*
2018	0.80 (0.67, 0.95)	0.010*	0.77 (0.65, 0.92)	0.004*
Urbanization	-	-	-	-
Urban	References	NA	References	NA
Suburban	1.42 (1.29, 1.58)	< 0.001*	1.45 (1.31, 1.61)	< 0.001*
Rural	4.99 (4.52, 5.50)	< 0.001*	5.27 (4.77, 5.83)	< 0.001*

Data are presented as odds ratio (95% CI). *CI* Confidence interval

^*^P-value < 0.05 was considered statistically significant after test

**Fig. 1:**
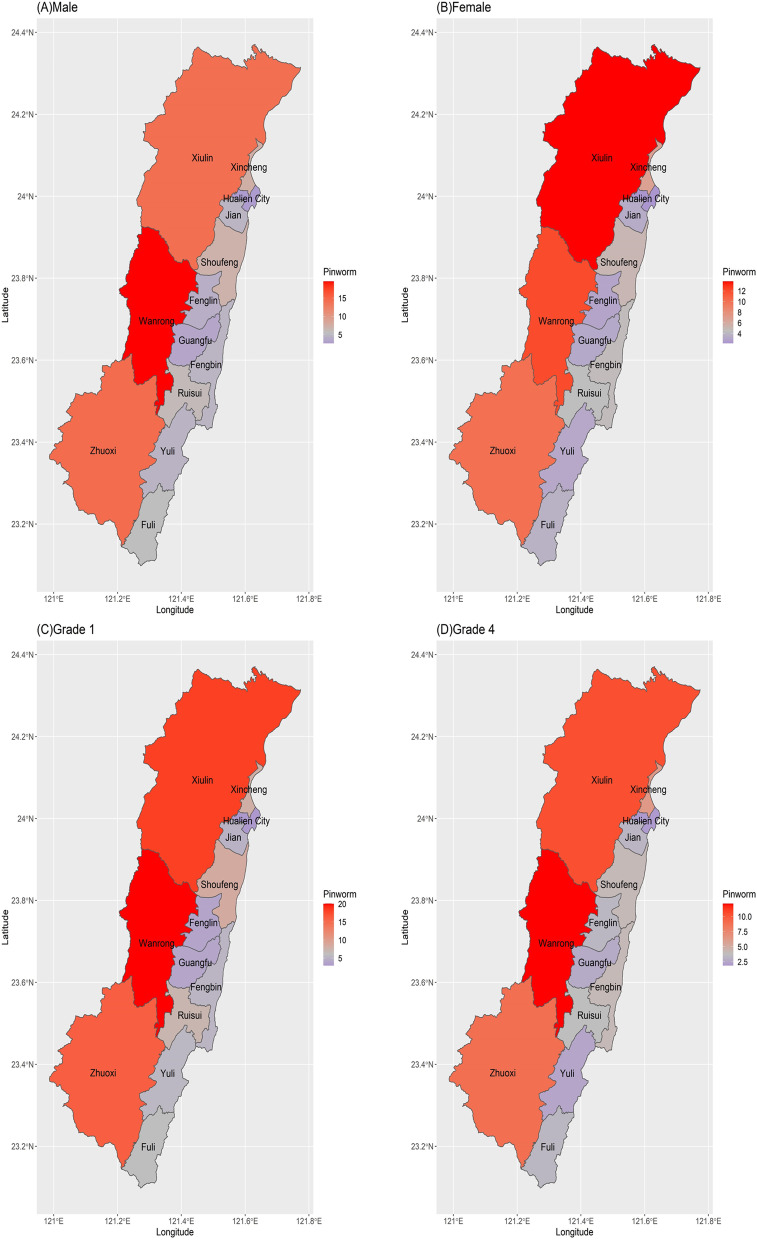
Prevalence of pinworm infection in different districts in Hualien. **A** Male. **B** Female. **C** Students of grade 1. **D** Students of grade 4

Figure 1 shows the prevalence of pinworm infection in different districts of Hualien. Hualien is located between tropical and subtropical zones. Pinworm infection rates were low in urban and suburban areas of Hualien, including Hualien City, Fenglin, Guangfu, and Yuli. Rural areas of Hualien included Xiulin, Wanrong, and Zhuoxi, which are near the mountains and at the junction of tropical and subtropical zones. Both sexes and students of grades 1 and 4 had a high pinworm infection rate in rural areas.

## Discussion

Pinworm infection is a major concern around the world with variable prevalence. Overall, a gradually decreased prevalence of pinworm infection has been noted in many countries, including Taiwan [4, 9, 10]. This study revealed the prevalence, time-trend analysis, and associated sociodemographic factors among school-age children in Hualien, Taiwan, from 2009 to 2018.

In this study, the overall prevalence of pinworm infection among school-age children was 4.4% from 2009 to 2018. In other studies, the prevalence of pinworm infection in Hualien was 3.1–4.2% according to different study populations and study periods [3, 10]. Generally, the overall prevalence of pinworm infection in Hualien was higher than that in Taiwan, which was about 2.4% (0.6–6.6%), as reported in a large-scale survey study [10]. These findings indicate that pinworm infection is still an important issue in Taiwan, especially in Hualien. Similarly, pinworm infection is also an important public health issue in some areas of resource-rich regions, although a declining infection rate was noted [5, 14, 18–20]. According to a study in Korea, pinworm infection is still detected in kindergartens and primary schools on the western and southern coastal islands of the Republic of Korea, with a prevalence rate of 0%–59.3% in different locations in 2000 [19]. In a retrospective study in greater Berlin, Germany, pinworm was detected in about 17.4% of the samples from 2007 to 2017 using the cellulose tape test [18].

In this study, we identified other risk factors for *enterobiasis* in children, including sex, age, and BMI. Sex-based differences in pinworm infection have been widely reported, and in our study, male subjects showed a significantly higher infection rate than female subjects. However, some studies revealed different results. A previous study in Taiwan found no significant sex-based difference in pinworm infection [2]. Another study conducted in Thailand found a higher risk of pinworm infection in female subjects than in male subjects [21]. Nevertheless, most studies revealed higher infection rates in males, which were in line with the findings of this study [3, 4, 7, 14, 19]. This phenomenon may be attributable to the fact that males have comparatively poor personal hygiene and indulge in more intimate contact activities with other children [4, 10, 19].

In this study, younger children in the first grade (6–7 years old) had a higher prevalence of *enterobiasis* than older children in the fourth grade (9–10 years old). This finding was consistent with previous studies that showed a higher risk of pinworm infection in preschool children than in school-age children, toddlers, and infants [2, 4, 8]. Although our study dataset did not include data on pinworm infection in toddlers and preschool children, an inverse trend between age and the prevalence of pinworm infection was still noted among school-age children. This phenomenon may be associated with improved personal hygiene in older children [2]. Another possible hypothesis was that the children in grade 4 with no pinworm infection may have a history of previous eradication of pinworm when they were in grade 1. However, there was no related data in this study to prove this hypothesis. Another potential reason may be related to the current screening method for pinworm infection in Taiwan, which entails the use of adhesive cellophane perianal swab for two consecutive days. Usually, this procedure is assisted by parents. Older children may be embarrassed to ask their parents for help and are likely to complete the procedure themselves. Therefore, the poor quality of perianal swabs may have introduced an element of bias in the detection of pinworm infection.

The negative correlation between BMI and pinworm infection was another interesting finding of this study. Overweight/obese BMI subjects had a significantly lower OR for *enterobiasis* than subjects with normal BMI. Similar findings have been reported in another study [22]. The potential reasons for the causality or association between high BMI and low pinworm infection rate are still uncertain. A previous study revealed that children with high BMI are more likely to spend more time watching television and show decreased physical activity [23], and the lifestyle of children with high BMI may decrease the risk of contact with people or the environment contaminated by pinworms. However, further study is required to clarify this hypothesis about the association between high BMI and low pinworm infection rate. Furthermore, underweight subjects in this study showed a trend for higher OR for *enterobiasis* infection than children in the normal BMI group but without statistical significance. Malnutrition is associated with intestinal parasitic infection. Furthermore, childhood developmental disorder and growth showed an association with pinworm infection [5, 24, 25]. The above-mentioned reasons may explain the higher risk of *enterobiasis* among underweight students in our study.

The non-obvious decline in the trend of pinworm infection rate in the rural areas of Hualien was an important finding of this study. This finding was similar to those of studies conducted in China, which reported that children in rural areas were at a higher risk of pinworm infection than those residing in urban areas [12, 26, 27]. However, there is no clear consensus on the association between urbanization and the prevalence of pinworm infection. In a study conducted in the Republic of Marshall Island, the prevalence of pinworm infection in urban areas was found to be higher than that in rural areas [8]. Another study in Taiwan also found that school-age children in rural areas are less likely to have pinworm infection than those residing in urban areas [1]. The reasons for the non-significant decrease in the trend of pinworm infection in remote areas of Hualien are likely to be multifactorial. First, low socioeconomic status was thought to be a risk factor for pinworm infection, and many studies have highlighted this condition [1, 2]. The rural areas in our study, including Xiulin, Wanrong, and Zhuoxi, have relatively low socioeconomic status, such as low educational level, low industrialization, and low family income. Furthermore, in Taiwan, there are significant urban–rural disparities with respect to the availability of health care, and medical resources are located mainly in the urban areas of Hualien. The above socioeconomic factors may be responsible for the persistently high prevalence of pinworm infection among school-age children in the remote areas of Hualien. Second, the high proportion of young children in rural populations in Hualien might be another reason for the persistently high prevalence of *enterobiasis* in these areas. According to the demographic data from the Hualien Civil Affairs Department, the three districts with the lowest population density were compatible with the rural areas in our study [28]. Despite the low population density, a higher percentage of children aged < 14 years in rural areas than in urban/suburban areas was a characteristic of Hualien according to the demographic data from the Hualien Civil Affairs Department [29]. Pinworm infection has been shown to be more common in young children aged 4 to 11 years [5, 21, 30]. Young children tend to play on the floor and show frequent nail-biting or finger-sucking behaviors and poor hand-washing compliance before meals; the above factors are known risk factors for pinworm infection [1, 4, 5, 8, 21]. Additionally, small schools might be related to the high prevalence of pinworm infection in the rural areas of Hualien. A large-scale survey of school-age children in Taiwan presented the association between the school size and the risk of pinworm infection [10]. Smaller schools (< 100 children) showed higher positivity rates of pinworm infection than larger schools (> 100 children). The authors concluded that children of different ages frequently use the same classroom due to the limited educational resources in these regions, which facilitates the transmission of pinworm due to the crowded environment. According to the data of the Taiwan Ministry of Education, most schools in the rural areas of Hualien areas are small schools, which corresponded to the areas with the highest prevalence of pinworm infection. In brief, the possible reasons for the persistently high prevalence of pinworm infection in rural areas included low socioeconomic status and the high percentage of young children, and small schools.

This study highlighted the fact that pinworm infection remains a vital public issue among school-age children in the rural areas of Hualien. Appropriate policy intervention for prevention of pinworm infection is important. Screening and eradication with mebendazole are the main policy interventions for pinworm infection in Taiwan, which led to a prominent declining trend of pinworm infection in recent decades. The screening groups among children were those in grades 1 and 4 owing to the overall low prevalence of pinworm infections in Taiwan in recent years. However, this policy does not seem to be sufficient to decrease the prevalence of pinworm infection in rural areas of Hualien. Therefore, it might be necessary to adjust the screening group policy in the hot pinworm zone. Further, we should make efforts to educate children in the red pinworm infection zones on sensitive issues such as adequate hand hygiene to prevent the spread of pinworm infection.

Some limitations of our study should be acknowledged. First, *enterobiasis* is well-known to be a multifactorial disease. Data pertaining to many other sociodemographic risk factors, such as the parental education levels, family income and living conditions, number of siblings, personal hygiene, long fingernails, and habitual frequent sucking of fingers, were not collected, leading to potential confounding in the final analysis [4, 8, 31]. Second, the data in this study were collected from the annual health examination of students in grades 1 and 4. Therefore, there were no data on other students except grades 1 and 4, and this may have introduced an element of selection bias. However, the large dataset used for this analysis is a study strength. Further studies are required for in-depth characterization of the trend of prevalence of pinworm infection in children in Hualien.

## Conclusion

Although overall pinworm infection among school-age children gradually decreased in Hualien from 2009 to 2018, pinworm infection remains a public health issue in the rural areas of Hualien. Related risk factors for pinworm infection included male, young age, and rural areas. Our study may help inform policy-level and public health interventions for prevention of *enterobiasis* in Taiwanese children.

## Data Availability

All data generated or analyzed during this study are included in this published article.

## Abbreviations

BMI: Body mass index
OR: Odds ratio
